# Adaptive, Fast Walking in a Biped Robot under Neuronal Control and Learning

**DOI:** 10.1371/journal.pcbi.0030134

**Published:** 2007-07-13

**Authors:** Poramate Manoonpong, Tao Geng, Tomas Kulvicius, Bernd Porr, Florentin Wörgötter

**Affiliations:** 1 Bernstein Center for Computational Neuroscience, University of Göttingen, Göttingen, Germany; 2 Department of Psychology, University of Stirling, Stirling, Scotland, United Kingdom; 3 Department of Electronics and Electrical Engineering, University of Glasgow, Glasgow, Scotland, United Kingdom; University College London, United Kingdom

## Abstract

Human walking is a dynamic, partly self-stabilizing process relying on the interaction of the biomechanical design with its neuronal control. The coordination of this process is a very difficult problem, and it has been suggested that it involves a hierarchy of levels, where the lower ones, e.g., interactions between muscles and the spinal cord, are largely autonomous, and where higher level control (e.g., cortical) arises only pointwise, as needed. This requires an architecture of several nested, sensori–motor loops where the walking process provides feedback signals to the walker's sensory systems, which can be used to coordinate its movements. To complicate the situation, at a maximal walking speed of more than four leg-lengths per second, the cycle period available to coordinate all these loops is rather short. In this study we present a planar biped robot, which uses the design principle of nested loops to combine the self-stabilizing properties of its biomechanical design with several levels of neuronal control. Specifically, we show how to adapt control by including online learning mechanisms based on simulated synaptic plasticity. This robot can walk with a high speed (>3.0 leg length/s), self-adapting to minor disturbances, and reacting in a robust way to abruptly induced gait changes. At the same time, it can learn walking on different terrains, requiring only few learning experiences. This study shows that the tight coupling of physical with neuronal control, guided by sensory feedback from the walking pattern itself, combined with synaptic learning may be a way forward to better understand and solve coordination problems in other complex motor tasks.

## Author Summary

The problem of motor coordination of complex multi-joint movements has been recognized as very difficult in biological as well as in technical systems. The high degree of redundancy of such movements and the complexity of their dynamics make it hard to arrive at robust solutions. Biological systems, however, are able to move with elegance and efficiency, and they have solved this problem by a combination of appropriate biomechanics, neuronal control, and adaptivity. Human walking is a prominent example of this, combining dynamic control with the physics of the body and letting it interact with the terrain in a highly energy-efficient way during walking or running. The current study is the first to use a similar hybrid and adaptive, mechano–neuronal design strategy to build and control a small, fast biped walking robot and to make it learn to adapt to changes in the terrain to a certain degree. This study thus presents a proof of concept for a design principle suggested by physiological findings and may help us to better understand the interplay of these different components in human walking as well as in other complex movement patterns.

## Introduction

When walking, humans can adapt quickly to terrain changes, and they can also learn to walk differently on different surfaces. This ability is known to us all when we quickly adapt our gait after having stumbled or more slowly devise different strategies for walking uphill, downhill, or on sand as compared with ice. Neurophysiological studies have revealed that these properties arise from a combination of biomechanics and neuronal control. For example, some walking animals (e.g., bears, dogs) may be able to stand up and walk a few steps, but will not be able to develop a stable gait because their biomechanical design (called here the biomechanical level) is inappropriate for this. Neuronal control, on the other hand, assures that different gaits can first be learned and then be quickly applied, for instance to adapt to the terrain.

**Figure 1:**
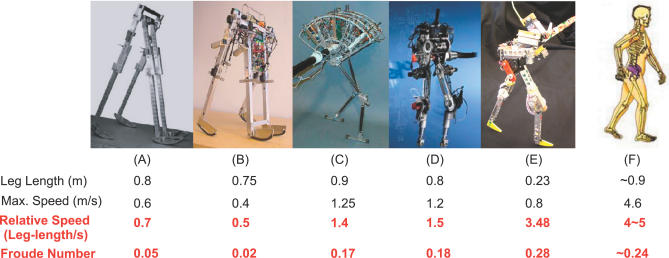
Relative Leg-Length and Maximum Relative Speed of Various Planar Biped Robots (A) A copy of McGeer's planar passive biped robot walking down a slope [77]. (B) “Mike,” similar to McGeer's robot, but equipped with pneumatic actuators at its hip joints. Thus it can walk half passively on level ground [77]. (C) “Spring Flamingo,” a powered planar biped robot with actuated ankle joints [78]. (D) Rabbit, a powered biped with four degrees of freedom and pointed feet [79]. (E) RunBot. (F) The world record for the fastest human's walking speed [80,81].

In the 1930s the Russian physiologist Bernstein [1–3] pointed out that the coordination of the cooperation within and between the different functional levels of the motor system, including controlled forms of motor learning, is a very difficult problem, e.g., due to the redundancy of effective movements (“The Bernstein Problem,” also discussed in [4]). Along this paradigm, Sporns and Edelman [5] proposed that a successful developmentally guided coordination between neuronal activity and the biomechanics of the musculoskeletal system can be achieved without determining a desired trajectory. Instead, it is based on variations of neuronal and biomechanical structures and is the result of somatic selection processes within brain circuits. The concept was applied to solve the arm-reaching problem, which was demonstrated with an artificial sensorimotor system. Mussa-Ivaldi and Bizzi [6] suggested a theoretical framework that combines some features of inverse dynamic computations with the equilibrium-point hypothesis for controlling a wide repertoire of motor behaviors also involving motor learning. They applied this to control the movement of a two-jointed robot arm with force fields as motor-primitives [6,7]. In the domain of dynamic legged locomotion control, Raibert [8] presented a series of successful hopping robots executing extremely dexterous and dynamic movements. The first of these robots is a single-legged running machine that works in two dimensions. It captures the feature of dynamic stability due to the carefully designed dynamics of the robot together with the use of simple feedback control. On the basis of these principles, Raibert and his collaborators extended their approach to a variety of machines using one, two, or four, legs, in two or three dimensions. Nakanishi et al. [9] reported one excellent example of biped locomotion control with motor learning. There, a central pattern generator (CPG) was employed to generate dynamical movement-primitives while the desired trajectories for walking behavior were learned by imitating demonstrated movement of humans. Nonetheless, some outstanding problems remain unsolved, in particular the problem of fast and adaptive biped walking based on self-stabilizing dynamic processes. Given that a biped has only one foot touching the ground during most of the time of a gait cycle, this poses huge difficulties for dynamic control, as the biped always tends to trip or fall. Thus, one particular objective of this article is to show that minimal adaptive neuronal control based on the reflexive mechanism [10] coupled with appropriate biomechanics can generate fast and adaptive biped walking gaits by a self-stabilizing process. As a result, our biped system can perform like a natural human walking (as shown by similar Froude numbers, see Figure 1) where the maximum walking speed is comparable to that of humans.

Neuronal walking control in general follows a hierarchical structure [11]. At the bottom level there are direct motor responses, often in form of a local, sometimes monosynaptic, reflex driven by afferent signals, which are elicited by sensors in the skin, tendons, and muscles—such as the knee tendon reflex. These sensor-driven circuits; which, following textbook conventions, we will call the *spinal (reflex) level;* can produce reproducible, albeit unstable gaits [12,13] and seem to play a more dominant role in nonprimate vertebrates [14] and especially in insects [15]. This level is often also augmented by CPGs in the spinal cord [14,16,17]. For example, Grillner [18,19] and others [20,21] have shown that generation of motor patterns as well as coordination of motor behavior in both vertebrates and invertebrates is basically achieved by CPGs which are in the central nervous system. Although CPGs provide the basis for generation of motor patterns, this does not mean that sensory inputs are unimportant in the patterning of locomotion. In fact, the sensory input is crucial for the refinement of CPG activity in response to external events.

Especially in humans, CPG functions seem to be less important for walking, and they had been hard to unequivocally verify [22] because they can strongly be influenced and, thus, superseded by sensory influences and by the activity of higher motor centers [14,23,24]. In general, higher motor centers modulate the activity of the spinal level, and their influence leads to our flexibility and adaptivity when executing gaits under different conditions. For example, inputs from peripheral sensors (e.g., eye, vestibular organ) can be used to adapt a gait to different terrains and also to change the posture of the walker, moving its body, to compensate for a disturbance. Reflexes also play a role at this level, called here the *postural (reflex) level,* but these long-loop reflexes [25,26] are always polysynaptic and can be much influenced by plasticity. Infants also use such peripheral sensor signals to learn the difficult task of adjusting and stabilizing their gaits [27,28], which many times amounts to learning how to avoid reflexes from earlier compensatory motor actions. The cerebellum seems to play a fundamental role in this type of motor learning for reflex-avoidance or reflex-augmentation [29]. A more specific discussion of this is presented in Materials and Methods. Beyond postural reflexes, we find ourselves at the level of motor-planning, which involves basal ganglia, motor cortex, and thalamus, with which this study is not concerned.

A suggested solution to the coordination problem (Bernstein Problem) invokes delegating control from higher to lower centers [4]. Central to this idea is the fact that the walking process itself leads to repetitive stimulation of the sensory inputs of the walker. As a consequence, at every step all neuro–mechanical components and their CPGs are retriggered [23], which could be used to control coordination. While an appealing idea, whose importance has been discussed recently by Yang and Gorassini [30], its applicability has so far not been demonstrated. In this study, we will try to show that sensor-driven control can be a powerful method to guide coordination of different levels in an artificial dynamic walker, and that this can also be combined with (neuronal) adaptivity mechanisms in a stable way.

To this end and following from the introduction, we assume that there are three important requirements for basic walking control: 1) biomechanical level—the walker requires an appropriate biomechanical design, which may use some principles of passive walkers to assure stability [31]. 2) spinal reflex level—it needs a low-level neuronal structure, which creates dynamically stable gaits with some degree of self-stabilization to assure basic robustness. 3) postural reflex level—finally, it requires higher levels of neuronal control, which can learn using peripheral sensing to assure flexibility of the walker in different terrains.

Fundamentally, these levels are coupled by feedback from the walking process itself, conveying its momentary status to different sensor organs locally in muscles and tendons and peripherally to the vestibular organ and the visual system as well as others as arising. At high walking speeds, cooperation of these three levels needs to take place very quickly and any learning also must happen fast. These demands for dynamic walking are currently impossible to fulfill with artificial (robot) walking systems, and the required tight interaction between levels embedded in a nested closed-loop architecture has not yet been achieved [31,32].

## Results/Discussion

In the following description, results are often being described alongside the structural elements from which they mainly derive, because this better reflects the tight intertwining of structure and function in this approach. Details on RunBot's structural elements are found in Materials and Methods.

**Figure 2:**
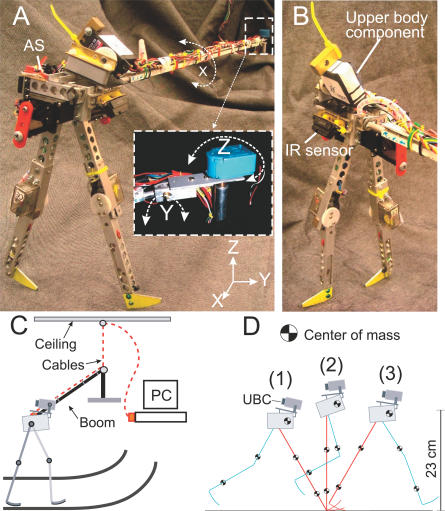
The Robot System (A,B) The planar dynamic robot *RunBot* with its active UBC. It is constrained sagitally by a boom freely rotating in three orthogonal axes. (C) The experimental setup of the RunBot system. (D) Illustration of a walking step of RunBot.

**Figure 3:**
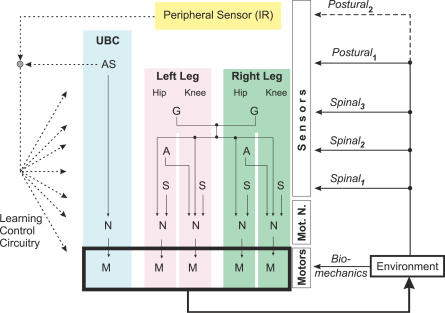
The Neuronal Control Structure of RunBot The different reflexive control levels of RunBot (solid lines). Also indicated is the influence of simulated plasticity (dashed lines), described in detail in Figure 11. The black box at the bottom represents RunBot's physical embodiment, colored boxes its neuronal control and sensor networks. Walking control arises from the interplay of the different sensori–motor loops (*spinal, postural*) implemented in RunBot together with its passive dynamic walking properties (*biomechanics*). *G*, ground contact sensor; *A*, stretch receptor for anterior extreme angle of the hips; *S*, local angle sensor neuron of hips and knees; *N*, motor neuron (*Mot.N.*); *M*, motor. For details of the agonistic–antagonistic wiring, see Figure 6.

The robot system “RunBot” (Figure 2) presented in this study has been developed during the last four years [33,34] and now covers these three levels of control (Figure 3), using few components and reaching a speed of up to 3.5 leg-length/s (see Video S1), which has so far not been achieved with other dynamic walkers. While still being a planar robot (supported in the sagittal plane), it is nonetheless a dynamic walking machine, which does not use any explicit gait calculation or trajectory control, but instead fully relies on its two neuronal control levels. As will be shown, at the postural reflex level the network can learn to use mechanisms of simulated synaptic plasticity, emulating the idea of learning to avoid a long-loop body-reflex.

### Biomechanical Level

**Figure 4:**
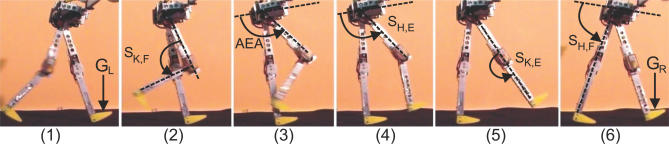
Series of Frames of One Walking Step At the time of frame (3), the stretch receptor (AEA signal) of the swing leg is activated, which triggers the extensor of the knee joint in this leg. At the time of frame (6), the swing leg begins to touch the ground. This ground contact signal triggers the hip extensor and knee flexor of the stance leg, as well as the hip flexor and the knee extensor of the swing leg. Thus, the swing leg and the stance leg swap their roles thereafter.

**Figure 5:**
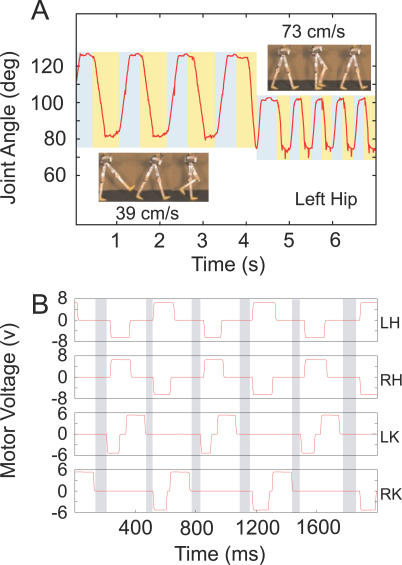
Real-Time Data of Walking Experiments (A) The joint angle of the left hip recorded during walking and changing speed on the fly. Parameters are changed greatly and abruptly for all extensor sensor thresholds of hip joints from Θ_*S*_*E*__ = 120.0 *deg* to 93.0 *deg* and for all hip motor neuron gain values from *g* = 1.55 to 3.0. This way speed changed from 39 cm/s (≈1.7 leg-length/s) to 73 cm/s (≈3.17 leg-length/s). At fast walking speed (73 cm/s, ≈3.17 leg-length/s), RunBot performs two steps per second, which is related to normal human walking speed [82]. Light blue areas indicate the swing phase of the left leg and light yellow areas are the stance phase. (B) Motor voltages directly sent from the leg motor neurons to the servo amplifiers while the robot is walking: LH, left hip; RH, right hip; LK, left knee; RK ,right knee. Gray areas indicate when all four motor voltages remain zero during some stage of every step cycle where the robot walks passively. Due to an appropriate weight of the limb together with the generated velocity, it leads to a momentum which is high enough to rotate the joint and swing the leg into the desired position although the motor voltages are zero, while the gear fiction will decrease the acceleration. Note that the controller of the RunBot system is implemented on a 2-GHz PC, and the data information is processed at a certain number of steps with the update frequency of 250 Hz.

RunBot has four active joints (left and right hips and knees), each of which is driven by a modified RC servo motor. It has curved feet allowing for rolling action and a lightweight structure with proper distribution of mass at the limbs (Figure 2D). The proper distribution of mass is calculated in the way that approximately 70% of the robot's weight is concentrated on its trunk where the parts of the trunk are assembled such that its center of mass is located forward of the hip axis. Furthermore, it has an upper body component (UBC), which can be actively moved to shift the center of mass backward or forward. Central to its mechanical design is the proper positioning of the center of mass, the effect of which is shown in Figures 2D and 4 during walking on flat terrain where the UBC is kept stable in its rearward position. One walking step consists of two stages. During the first stage (steps (1) and (2) shown in Figure 2D, compare with steps (1)–(3) shown in Figure 4), the robot has to use its own momentum to rise up on the stance leg. When walking slowly, this momentum is small and, hence, the distance the center of mass has to cover in this stage should be as small as possible, which can be achieved by a low and slightly forward placed center of mass similar to humans [35]. In the second stage (steps (2) and (3) shown in Figure 2D, compare with steps (3)–(6) shown in Figure 4), the robot just falls forward naturally and catches itself on the next stance leg [36]. Hence, RunBot's design (see Figure 2) relies quite strongly on the concepts of self-stabilization of gaits in passive walkers [31]. This property is emulated by the lowest loop (Biomechanics [37]) in Figure 3. RunBot's passive properties are also reflected by the fact that during one quarter of its step cycle all motor voltages remain zero, as shown in Figure 5B (gray areas). A detailed simulation analysis of the stability properties of RunBot is given in [33,34].

**Figure 6:**
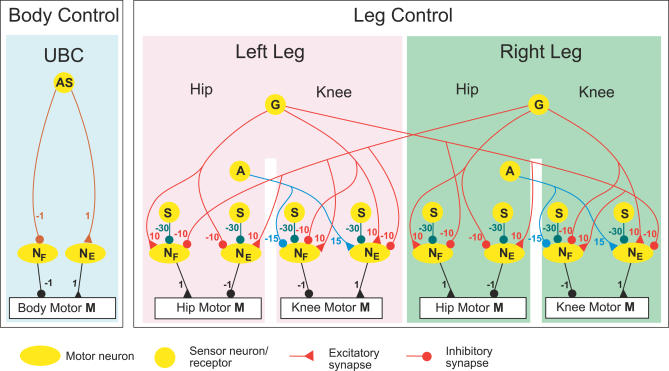
The Reflexive Neuronal Network It consists of two distributed neural modules for leg and body control. The connection strengths (color lines) are indicated by the small numbers. *A* refers to the stretch receptors for *AEA* of the hips and *G* to the ground contact sensor neurons of the feet. *N_F_*(*N_E_*) refers to flexor (extensor) motor neuron of the body and leg. *S* represents the angle sensor neurons of each joint. The *AS* neuron is used to trigger the UBC reflex.

### Spinal Reflex Level

Figure 3 represents the basic neuronal control structure of RunBot. The right, uncolored side shows the general signal flow from sensors via motor neurons (Mot.N.) to the motors involving several closed loops. To reduce computational overhead, we designed the neuronal control network (left side) using only standard sigmoid, Hopfield-type neurons (see Materials and Methods). The circuitry in general consists of an agonist–antagonist control structure for hips and knees with flexor and extensor components, a dichotomy which we have, for clarity, omitted in Figure 3 (for details of the agonist–antagonist connectivity, see Figure 6). Its motor neurons *N* are linear and can send their signals unaltered to the motors *M*.

Furthermore, there are several local sensor neurons which by their conjoint reflex-like actions trigger the different walking gaits. We distinguish three local loops. Joint control arises from sensors *S* at each joint (compare Figure 4), which measure the joint angle and influence only their corresponding motor neurons (*Spinal*_1_). Interjoint control is achieved from sensors *A*, which measure the anterior extreme angle (AEA, Figure 4) at the hip and trigger an extensor reflex at the corresponding knee (*Spinal*_2_). Leg control comes from ground contact sensors *G* (compare Figure 4), which influence the motor neurons of all joints in a mutually antagonistic way (*Spinal*_3_).

In addition, there is the control circuit for the UBC (Figure 3). This circuit represents a long-loop reflex (*Postural*_1_), and its accelerometer sensor (AS) is also involved in controlling plasticity within the whole network. Here we first describe its pure reflex function prior to learning. The UBC is controlled by its flexor and extensor motor neurons *N_F_*,*N_E_*, driven by the activity of one AS neuron. (Indexing of variables in this article follows this structure: body-level (UBC = B, left-leg = L, right-leg = R); leg level (hip = H, knee = K); joint level (flexor = F, extensor = E). In general, indices are omitted below the last relevant level, i.e., *S_L,H,E_* applies to the extensor of the hip of the left leg, whereas *S_L,H_* would apply to flexor *and* extensor of the hip of the left leg.

**Figure 7:**
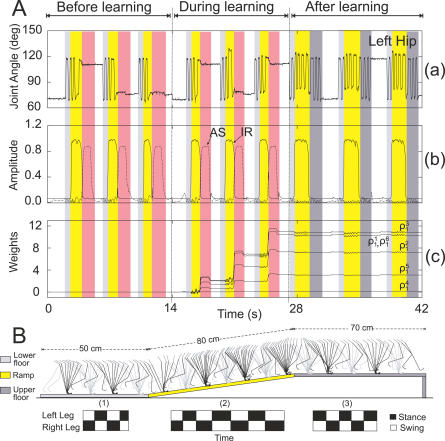
Adaptive Walking Experiments (A) The real-time data of left hip angle (a), reflexive AS and predictive IR signals (b), and plastic synapses *ρ*_1_ (c) in three situations where there was no learning for walking up a slope at the beginning. Learning was switched on at 14 s (dashed line). After that, learning self-stabilized and ended at about 28 s (dashed line). The data was recorded while RunBot was initially walking from a lower floor (light gray areas) to an upper floor (dark gray areas) through a ramp (yellow areas). Note that red areas depict the situation where RunBot falls backward and white areas where RunBot was manually returned to the initial position. In this experiment, RunBot can manage to walk on an 8° ramp after three falls, which is approximately 14 s of learning time. (B) Stick diagram of RunBot walking on different terrains where black (gray) shows the right (left) leg. RunBot started to walk on a level floor, then on an 8° ramp, and finally it continued again on a level floor. Average walking speed was about 50 cm/s (≈2.17 leg-length/s). The interval between any two consecutive snapshots of all diagrams is 67 ms. In this walking experiment, we set *g^max^* to 2.2. The lower diagrams show the walking step of RunBot corresponding to each walking condition above. During the swing phase (white blocks), the respective foot has no ground contact. During the stance phase (black blocks), the foot touches the ground. As a result, one can recognize the different gaits between walking on a level floor, (1) and (3), and walking up the ramp, (2).

**Figure 8:**
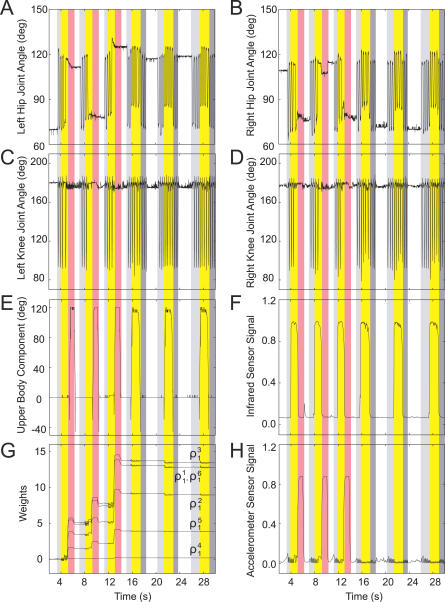
Real-Time Data of All Leg Joints and Body Motion The data was recorded while RunBot was initially walking from a lower floor (light gray areas) to an upper floor (dark gray areas) through a ramp (yellow areas). Red areas represent situations where RunBot falls backward and white areas where RunBot was manually returned to the initial position. In this experiment, RunBot can manage to walk on an 8° ramp after three falls. (A–D) Show the left and right joint angles at all situations. (E) Shows the posture of the UBC where 0° means leaning backward while 120° mean leaning forward. (F,H) Show the predictive (IR) and reflexive (AS) signals, respectively. The growing synaptic strengths (compare Figure 13A) during the learning phase are represented in (G).

On flat terrain, AS is inactive and the flexor is activated to lean the body backward while the extensor is inhibited. This situation is reverted when a strong signal from the AS exists, which happens only when RunBot falls backward (see learning experiments in Figures 7 and 8). This will trigger a leaning reflex of the UBC.

**Figure 9:**
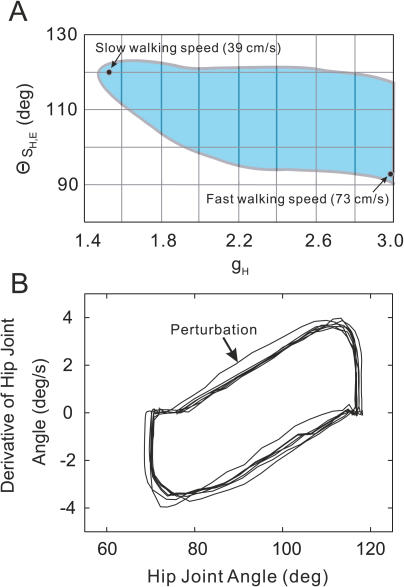
The Stable Domain of the Controller Parameters and the Stability Analysis (A) The shaded area is the stable domain of the controller parameters (Θ_*S_*H*,*E*_*_ , *g_H_*), where stable gaits will appear in experiments performed with the real robot. (B) Limit cycles in phase plane dΘ_*S*_*H*__/dt, Θ_*S*_*H*__ for walking on a flat floor (Θ_*S*_*H*,*F*__ = 78.0°, Θ_*S*_*K*,*F*__ = 115.0°, Θ_*S*_*H*,*E*__ = 105.0°, Θ_*S*_*K*,*E*__ = 175.0°, *g_K_* = 1.8 and *g_H_* = 2.2). It shows that after being perturbed, the walking gait returns to its limit cycle quickly in only a few steps. Note that RunBot can neither detect the disturbance nor adjust any parameters of its controller to compensate for it.

This way, different loops are implemented, all of which are under sensory control, which assures stability of walking within wide parameter ranges. In Figure 9A, we show the stable domain for the two most sensitive parameters *g_H_* and 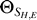 . Within the blue area, a wide variety of different gaits can be obtained, two of which (marked) are shown in Figure 5. To analyze the dynamical stability of RunBot, which follows a cyclic movement pattern, the Poincare-map method [38] is employed, because our reflexive controller exploits natural dynamics for the robot's motion generation, and not trajectory planning or tracking control. A simulation analysis of our robot system with Poincare maps has been shown in our previous study [34]. Here we present the stability analysis in a real walking experiment (Figure 9). In Figure 9B, we show a perturbed walking gait where the bulk of the trajectory represents the normal orbit of the walking gait, while the few outlying trajectories are caused by external disturbances induced by small obstacles such as thin books (less than 4% of robot size) obstructing the robot path. After a disturbance, the trajectory returns to its normal orbit soon, demonstrating that the walking gaits are stable and to some degree robust against external disturbances. Here, robustness is defined as rapid convergence to a steady-state behavior despite unexpected perturbations [39]. That is, the robot does not fall and continues walking.

**Figure 10:**
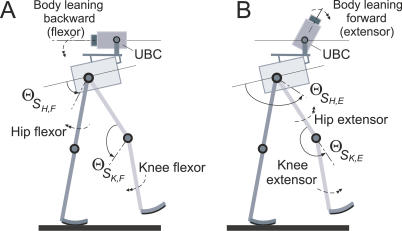
Control Parameters for the Joint Angles (A) Flexor angles. (B) Extensor angles. Θ_*S*_*E*__ (Θ_*S*_*F*__) indicates the threshold of the sensor neuron for extensor (flexor). H, hip; K, knee.

Furthermore, the intrinsic robustness of the RunBot system makes parameter fine-tuning unnecessary, which can be judged from Figure 5A. Here we show that it is possible to immediately switch manually from a slower walking speed of 39 cm/s (≈1.7 leg-length/s) to a faster one of 73 cm/s (≈3.17 leg-length/s) (see Video S1). This result has been achieved by abruptly and strongly changing two parameters: the threshold of the local extensor sensor neurons of hip joints (see Figure 10, 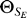 ) and the gain *g_H_* of hip motor neurons. The dynamic properties of RunBot allow doing this without tripping it, and speed is almost doubled. Such quick and large changes in walking speed are no problem for humans but difficult if not impossible for existing biped robots. The self-stabilization against such a strong change demonstrates that RunBot's neuronal control parameters, analyzed in [33,34], are not very sensitive and that a wide variety of stable gaits (see Figure 9A) can be obtained by changing them. The leg motor signals shown in Figure 5B demonstrate that during about one quarter of RunBot's step cycle all leg motors are inactive (zero-voltage), making RunBot a passive walker during this time.

**Figure 11:**
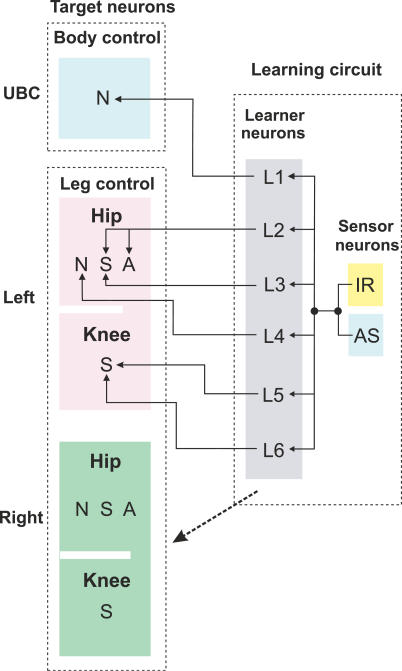
Postural Neuronal Control Connections between learner neurons and target neurons of the right leg, which are identical to those of the left leg, are not shown (see text and also Materials and Methods for details).

To compare the walking speed of various biped robots whose sizes are quite different from each other, we use the relative speed, which is speed divided by the leg-length. Maximum relative speeds of RunBot and some other typical planar biped robots (passive or powered) are listed in Figure 1. We know of no other biped robot attaining such a fast relative speed. Moreover, the world record for human walking is equivalent to about 4.0–5.0 leg-length/s. So, RunBot's highest walking speed is comparable to that of humans. In general, the Froude number *Fr* is used to describe the dynamical similarity of legged locomotion over a wide range of animal sizes and speeds on earth [40]. It can be determined by *Fr* = *v* ^2^/*gl* where *v* is the walking speed, *g* gravity, and *l* leg-length. Figure 1 also gives *Fr* for different designs, where *Fr* of RunBot and humans are quite similar.

### Postural Reflex Level

For the postural level, we have implemented a long-loop body reflex at the UBC, triggered by a strong backward lean as described above. This reflex can be changed by learning, which will also influence several other network parameters to adapt the gait. The learning goal is to finally avoid the leaning reflex and at the same time to learn changing gait parameters in an appropriate way to prevent RunBot from falling. This requires an adaptive network of six more neurons (Figure 11) which converge onto different target neurons at the spinal-level network, effectively changing their activation parameters (see Materials and Methods). RunBot's task was to learn walking up a ramp and then continuing on a flat surface. Without gait and posture change, the robot can walk on slopes of only up to 2.5° [34]. Leaning the UBC forward and changing several gait parameters, RunBot manages about 8.0°. With a larger UBC mass, even steeper slopes (up to 13.0°) can be tackled, while walking down slopes can be also achieved in the reverse way with an appropriate gait. This is achieved by learning which is based on simulated plasticity.

It is known that neurons can change their synaptic strength according to the temporal relation between their inputs and outputs. If the presynaptic signal arrives before the postsynaptic neuron fires, such a synapse gets strengthened, but it will get weakened if the order is reversed. Hence, this form of plasticity depends on the timing of correlated neuronal signals (STDP, spike timing-dependent plasticity [41]). In neurons with multiple inputs, such a mechanism can be used to alter the synaptic strengths, through heterosynaptic interactions, according to the order of the arriving inputs. Formally, we have *v* = Σ*ρ_i_u_i_* as the neurons output driven by inputs *u_i_*, where synapses *ρ* get changed by differential Hebbian learning using the cross-correlation between both inputs *u*_0_ (the AS) and *u*_1_ (the IR (infrared) sensor) [42] (see also Materials and Methods). As a consequence, if an *early* input signal is followed by a *later* input, where the later one drives the neuron into firing, then the early input will get strengthened.

### Adaptive Walking Experiments

We make use of this type of sequence learning in adaptive walking experiments on different terrains, where RunBot was configured with a parameter set suitable for walking on a flat surface and learned to tackle an 8° ramp, which it manages after about three to five falls (see Figure 7A and Video S2). Its change in walking pattern after starting to climb the ramp is shown in Figure 7B. It takes about two steps on the slope for the machine to find its new equilibrium, which results in a slower stride up the slope as compared with flat terrain. The slowing down can be explained by the gravitational pull. Stride length, however, is shorter, and RunBot takes about seven steps on the slope, which is 80 cm long, while for the same distance it uses six steps on flat ground. Shortening the step size is similar to human behavior and is a result of the different parameters used for climbing together with the changed gravitational pull. Returning to the initial gait when reaching the top is faster and happens immediately. Note that RunBot's intrinsic stability can also be demonstrated by the fact that it will always succeed in walking up the slope, after having learned the new parameters, regardless of its starting point and independent of the positioning of the legs (as long as this allows making the first step).

A complete set of curves taken from RunBot similar to Figure 7A but from a different experiment is presented in Figure 8. Every “spike” in the top panels (Figure 8A–8D) represents one step. Figure 8F and 8H show that the IR signal does indeed come earlier as compared with the AS signal. This is also visible in Figure 8E, where the leaning reaction first coincides with the AS signal and only after learning comes together with the IR signal. Figure 8G shows all synaptic weights *ρ*_1_ that grow with a different rate (*μ*) and stabilize at different values. Small glitches in the weights observed after the last fall (see for example at about 22 s) arise from the fact that the AS sensor will always produce a little bit of noise, which leads to a weak correlation with the IR signal and to minor weight changes. Note that weights will only change strongly again if the AS signal produces another strong response; hence, in the case that the robot falls again. Thus, learning is stable as soon as the AS-triggered reflex is being avoided, but will set in anew if the robot should fall again.

As demonstrated in Figures 7 and 8, on approaching the ramp, RunBot's IR sensor will sense the slope *early*, but initially the IR sensor signal converges with zero strength at the network and goes unnoticed. As a consequence, RunBot will begin walking up the ramp with a wrong set of gait parameters and will eventually fall, leading to a *later* signal at the AS. The AS signal triggers the leaning reflex of the UBC together with the gait adaptation, but too late. However, the *early* IR sensor signal and the *later* AS signal converge at the same neurons, and due to simulated plasticity the synapses from the *early* IR inputs will grow. As a consequence, after some learning, the postural control network (see Figure 11) will receive nonzero input as soon as the IR sensor becomes active, RunBot will perform the leaning action earlier, and its gait will be changed in time. The used differential Hebbian learning rule has the property that learning will stop when the late input (AS signal) is zero [42], which is the case as soon as the reflex has successfully been avoided and the robot does not fall anymore. Hence, we obtain behavioral and synaptic stability at the same time without any additional weight-control mechanisms.

Recent studies on biped robots have emphasized the importance of the biomechanical design by focusing on so-called passive dynamic walkers, which are simple devices that can walk stably down a slope [43]. This is achieved only by their mechanical design. Adding actuators to their joints may allow these robots to walk also on a level surface or even uphill. The developed gaits are impressively human-like [31], but these systems cannot easily adapt and/or change their speed. More traditionally, successful robot-walkers have been built based on precise joint-angle control, using mainstream control paradigms such as trajectory-based methods [44], and some of the most advanced robots are constructed this way; e.g., *ASIMO* [45], *HRP*2 [46], *JOHNNIE* [47], and *WABIAN* [48]. It is, however, difficult to relate these machines to human walking, because closed-loop control requires highly precise actuators unlike muscles, tendons, and human joints, which do not operate with this precision. Furthermore, such systems require much energy, which is in conflict with measured human power consumption during walking or running [36,49,50], and their control is non-neuronal. Neuronal control for biped walking in robots is usually achieved by employing CPGs [51,52], which are implemented as a local oscillator under limited sensor control. Furthermore, if adaptive mechanisms are employed [32,53], then conventional techniques from machine learning are used, which are not directly related to neuronal plasticity. The controller described in [54] is also based on the concept of CPGs where the trajectory of *each* joint is modeled by a specific oscillator. These are globally synchronized through sensory information (e.g., ground reaction force) together with the robot dynamics, instead of being partially autonomous. The method does not start with generated limb patterns or a formal proof of stability as used in trajectory-based methods. By contrast, the model in Morimoto et al. has been designed and then tuned to obtain the desired effect. As a consequence of its simplicity, one can add more feedback in the control loop, or modify the generated trajectories without having to restart a global optimization process.

The strategy pursued here is to some degree related—RunBot also relies on sensory feedback to synchronize its components, which are arranged in nested loops ([8,55], see Figure 3), but without the help of CPGs. Instead, we achieved tight coupling of the different levels of physical and neuronal control via feedback from the walking processes itself, which conveys its momentary status to different sensors; locally at the joints/legs and peripherally to our very simple simulated “vestibular organ” (AS) and “visual system” (IR). This structure made it possible to also implement a fast learning algorithm, which is driven by peripheral sensors but influences all levels of control; explicitly by augmenting neuronal parameters and implicitly at the biomechanical level by the resulting new walking equilibrium. The idea of downward-delegating coordination control, where local levels maintain a high degree of sensori-driven autonomy [3,4], could thereby be implemented and tested.

We believe that this demonstration is the major contribution of the current study. It shows that complex behavioral patterns result from a rather abstract model for locomotion and gait control consisting of a simple set of nested loops. Much of the biologically existing complexity has been left out. This especially should stimulate further biological investigations because little is known about how a possible Bernstein mechanism is actually implemented in humans for locomotion and gait control. The existing data in this field is plentiful and diverse, but often conflicting evidence exists for certain subfunctions. This may be due to neglect of context within which a certain dataset has been obtained. Thus, given the rich existing data, a better understanding of human locomotion would probably require a focus of new research on abstractions and synthesis trying to combine the different strands into a closed form picture and only carefully extending the existing datasets. This may also help to resolve the existing conflicts because synthesis will enforce context.

Highly adaptive and flexible biped walking will certainly require additional mechanisms beyond those implemented here; for example, augmenting neuronal control via internal models of the expected movement outcome (“efferent copies” [56]) and/or adding intrinsic loops for CPG-like functions [14,22]. The results presented here, however, suggest that the employed nested-loop design remains open to such extensions bringing the goal of fully dynamic and adaptive biped walking in artificial agents a little bit closer.

## Materials and Methods

### Mechanical setup of RunBot (biomechanical level).

RunBot is 23 cm high, with a foot-to-hip joint axis (see Figure 2). Its legs have four actuated joints: left hip, right hip, left knee, and right knee. Each joint is driven by a modified RC servo motor where the built-in Pulse Width Modulation (PWM) control circuit is disconnected, while its built-in potentiometer is used to measure the joint angles. A mechanical stopper is implemented on each knee joint to prevent it from going into hyperextension, similar to the function of human kneecaps. The motor of each hip joint is a HS-475HB from Hitec. It weighs 40 g and can produce a torque up to 5.5 kg·cm. Due to the use of the mechanical stopper, the motor of the knee joint bears a smaller torque than the hip joint in stance phases, but must rotate quickly during swing phases for foot clearance. Therefore, we use a PARK HPXF from Supertec on the knee joints, which has a light weight (19 g), but is fast with 21 rad/s. Thus, approximately 70% of the robot's weight is concentrated on its trunk, and the parts of the trunk are assembled in a way that its center of mass is located forward of the hip axis.

**Figure 12:**
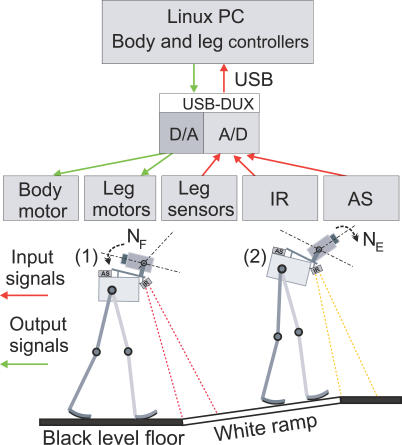
Schematic Setup of the RunBot System Leg sensors consist of joint angle sensors and ground contact switch sensors, leg motors are the motors of the left and right hip and knee joints, and the body motor indicates the motor of the UBC. IR and AS stand for infrared and accelerometer sensors, respectively. The detection range of the IR sensor for slope sensing is shown in the lower picture. Note that the red ray of the IR sensor indicates that the sensor gives a high output signal while the yellow ray means a low signal. Hence, the sensor responds more strongly to the white ramp.

RunBot has no actuated ankle joints, resulting in very light feet and efficiency for fast walking. Its feet were designed to have a small circular form (4.5 cm long), whose relative length, the ratio between the foot-length and the leg-length, is 0.20, less than that of humans (approximately 0.30) and that of other biped robots (powered or passive, see discussion in [31]). Each foot is equipped with a switch sensor to detect ground contact events. The mechanical design of RunBot has some special features; for example, small curved feet and a properly positioned center of mass that allow the robot to perform natural dynamic walking during some stage of its step cycles. Hip and knee joints are driven by output signals of the leg controller (running on a Linux PC) through a DA/AD converter board (USB-DUX). The USB-DUX provides eight input (A/D) and four output (D/A) channels, and it has the update frequency of 250 Hz. The signals of the joint angles and ground contact switches are also digitized through this board for the purpose of feeding them into the leg controller (compare Figure 12).

**Figure 13:**
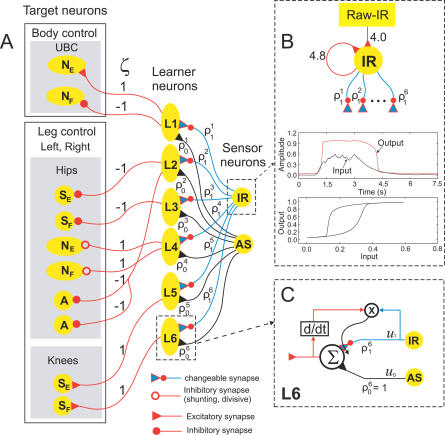
Adaptive Neuronal Controller with Learning Mechanism (A) The adaptive neuronal network. The excitatory synapses r_0_ projecting from the AS neuron to the learner neurons (black triangles) are all set to 1. While the changeable synapses r_1_ projecting from the IR neuron to the learner neurons are initially set to 0, they will grow during learning. Eventually, each of them will have converged to different values when learning stops. (B) Recurrent neural preprocessing of the IR signal configured as a hysteresis element. The curves below show the IR signal before preprocessing (*Input*) and the output signal after preprocessing (*Output*). The bottom curve presents the hysteresis effect between input and output signals. In this situation, the input varies between 0 and 0.6. Consequently, the output will gradually show high activation (≈1.0; meaning that RunBot approaches the ramp) when the input increases to values above 0.25. On the other hand, it will gradually show low activation (≈0.0; meaning that no ramp is detected) when the input decreases below 0.15. (C) Learning mechanism (see text for details). Note that all learner neurons have the same learning mechanism.

To extend its walking capabilities for walking on different terrains, for example level floor versus up or down a ramp, one servo motor with a fixed mass, called the UBC, is implemented on top. The UBC has a total weight of 50 g. It leans backward (see Figure 2A) during walking on a level floor, and this position is also suitable for walking down a ramp [57], and it will lean forward (see Figure 2B) when RunBot falls backward, and when it has successfully learned to walk up a ramp. The corresponding reflex is controlled by an AS, see Figure 2. The AS is installed on top of the right hip joint. In addition, one IR sensor is implemented at the front part of RunBot (see Figure 2) pointing downward to detect ramps (see Figure 12). Here, the IR sensor serves as a simple vision system, which can distinguish between a level floor with black color and a painted ramp with white color. This sensory signal is used for adaptive control. In our setup, the AS and IR signals are in parallel-feed to the USB–DUX for digitalization, providing them to the leg and body controllers afterward. The scheme of our setup is shown in Figure 12.

We constrain RunBot in the sagittal plane by a boom of one meter length. RunBot is attached to the boom via a freely rotating joint in the *x-*axis, while the boom is attached to the central column with freely rotating joints in the *y* and *z* axes (see Figure 2A). With this configuration, the robot is in no way being held up or suspended by the boom, and its motions are only constrained on a circular path. Given that the length of the boom is more than four times the height of RunBot, the influence of the boom on RunBot's dynamics in the sagittal plane is negligible. In addition, by way of an appropriate mounting (see Figure 2C), cabling also does not influence the dynamics of the walker. As shown here, the mechanical design of RunBot has the following special features that distinguish it from other powered biped robots and that facilitate high-speed walking and exploitation of natural dynamics: (a) small, curved feet allowing for rolling action; (b) unactuated, hence light, ankles; (c) lightweight structure; (d) light and fast motors; (e) proper mass distribution of the limbs; and (f) properly positioned mass center of the trunk.

This is a common strategy toward fast walking which facilitates scalability and is, thus, also present in other large robots, as in the new design of LOLA, the followup to JOHNNIE ([58], personal communication).

In general, scalability can be achieved by dynamic similarity [40,59]; for example, reflected in the same Froude number. Hence, by using similar design principles together with appropriate simulations (see, for example, [34]), one can gradually upscale such designs. This justifies the cost-effective small RunBot architecture from which basic principles can be extracted. Clearly, difficulties are expected to arise when introducing more degrees of freedom, but this reflects a true change in the system, not just an upscaling.

### The reflexive neuronal controller (spinal reflex level).

The reflexive neuronal controller of RunBot is composed of two neural modules: one is for leg control and the other for body control. The UBC and the peripheral sensors (*AS*, *IR*) are mounted on the rump of RunBot. Both controllers have a distributed implementation, but they are indirectly coupled through the biomechanical level; this way, the neural control network driven by the sensor signals will synchronize leg and body movements for stable locomotion.

*Leg control.* Leg control of RunBot consists of the neuron modules local to the joints, including motor neurons *N* and angle sensor neurons *S,* as well as a neural network consisting of hip stretch receptors *A* and ground contact sensor neurons *G* (see Figure 6), which modulate the motor neurons. Neurons are modelled as nonspiking neurons (Hopfield-type neurons) simulated on a Linux PC with an update frequency of 250 Hz, and communicated to the robot via the USB–DUX (see Figure 12). Nonspiking neurons have been used to increase the speed of network operations. Connection structure and polarity are depicted in Figure 6.

The top part of Figure 6 shows the ground contact sensor neurons *G*, which are active when the foot is in contact with the ground (see Figure 4). Its output changes according to:

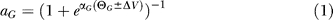
Where Δ*V* equals *V_R_* − *V_L_*, computed by the output voltage signals from switch sensors of the right foot *V_R_* and left foot *V_L_*, respectively, used with a plus sign in Equation 1 for the left and with a minus sign for the right ground contact sensor. Furthermore, Θ*_G_* are thresholds and *α_G_* positive constants.

Beneath the ground contact sensors, we find stretch receptor neurons *A* (Figure 6). Stretch receptors play a crucial role in animal locomotion control. For example, when the limb of an animal reaches an extreme position, its stretch receptor sends a signal to the controller, resetting the phase of the limbs [10]. There is also evidence that phasic feedback from stretch receptors is essential for maintaining the frequency and duration of normal locomotive movements in some insects [37].

Different from other designs [10,60], our robot has only one stretch receptor on each leg to signal the *AEA* of its hip joint (see Figure 4). Furthermore, the function of the stretch receptor on our robot is only to trigger the extensor motor neuron on the knee joint of the same leg (compare Figure 4), rather than to implicitly reset the phase relations between different legs, as, for example, in the model of Cruse [10].

The outputs *a_A_* of the stretch receptor neurons *A* for the left and the right hip are:

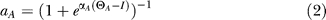
where *I* denotes the input signal of the neuron, which is the real time angular position of the hip joint *φ,* and *α_A_* is a positive constant. The hip anterior extreme angle Θ*_A_* depends on the walking pattern, for example Θ*_A_* = 105.0 *deg* for walking on a level floor, while it will be modified according to a learning rule for walking up a ramp described in the next section. This model is inspired by a sensor neuron model presented in [61].

At the joint level (Figure 6), the neuron module is composed of two angle sensor neurons (*S_E_*, *S_F_*) (see Figure 4) and the motor neurons (*N_E_*, *N_F_*) they contact (see Figure 6).

Whenever its threshold is exceeded, the angle sensor neuron *S* directly inhibits the corresponding motor neuron. This direct connection between angle sensor neurons and motor neurons is inspired by monosynaptic reflexes found in different animals [62] and also in humans [63].

The model of the angle sensor neurons *S* is similar to that of the stretch receptor neurons *A* described above. The angle sensor neurons change their output according to:

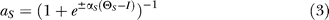
where *I* is an input signal, which is the real time angular position *φ* obtained from the potentiometer of the joint. Θ*_S_* is the threshold of the motor neuron and *α_S_* a positive constant. The plus sign is for the extensor angle sensor neuron 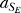 , and the minus sign is for the flexor angle sensor neuron 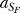 .

These three sensor signals (*G*,*A*,*S*) converge on the motor neurons *N* with different polarity, as shown in Figure 6. Some signals connect between joints or between legs, which assures correct cross-synchronization.

The motor neuron model is adapted from [60]. The state and output of each extensor and flexor motor neuron are governed by Equations 4 and 5 [64]:

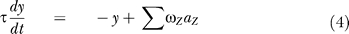


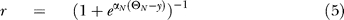
where *y* represents the mean membrane potential of the neuron. Equation 5 is a sigmoidal function that can be interpreted as the neuron's short-term average firing frequency, *α_N_* is a positive constant. Θ*_N_* is a bias constant that controls the firing threshold. τ is a time constant associated with the passive properties of the cell membrane [64]. *ω_Z_* represents the connection strength from the sensor neurons and stretch receptors to the motor neuron (Figure 6). The value of *a_Z_* represents the output of the sensor neurons and stretch receptors that contact this motor neuron (e.g., *a_S_*, *a_A_*, *a_G_*, etc.).

The voltage of the motor *U* in each joint is determined by:

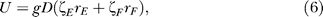
where *D* represents the magnitude of the servo amplifier, which is predefined by the hardware with a value of 3.0 on RunBot and *g* stands for the software-settable output gain of the motor neurons in the joint. The variables *ζ_E_* and *ζ_F_* are the signs for the motor voltage of extensor and flexor in the joint, being +1 or −1, depending on the hardware of the robot (compare Figure 6), and *r_E_* and *r_F_* are the outputs of the motor neurons.

*Parameters for leg control.* RunBot is quite robust against changes in most of its parameters (see details in [33]). Therefore, most parameters could be manually tuned by a few experiments supported by simulations (see [33]). We set: 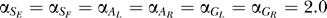 , but *α_N_* = 1.0, which assures a quick response of the corresponding neurons.

The threshold of the sensor neurons for the extensor (flexor) in the neuron module roughly limits the movement range of the joint and effects stability of locomotion on the different terrains. For instance, for walking on a level floor, we choose 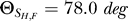 , 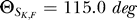 , 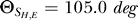 , and 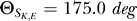 (compare Figure 10), which is in accordance with observations of normal human gaits [65]. The movements of the knee joints are needed mainly for timely ground clearance. After some trials, we set the gain of the motor neurons in the knee joints to *g_K_* = 1.8. Furthermore we set *g_H_* = 2.2.

The threshold of the stretch receptors is simply chosen to be the same as that of the sensor neurons for the hip extensor, 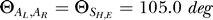 . With these parameters, we obtain a walking speed of about 50 cm/s (≈2.17 leg-length/s). However, the walking speed of RunBot can be increased up to 80 cm/s (≈3.48 leg-length/s) when *g_H_* is increased, while 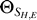 is decreased (described more details in [33]).

Note that for walking up a ramp, seven parameters (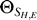 , 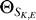 , 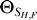 , 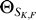 , 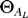 , 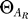 , and *g_H_*) will be modified by the synaptic plasticity mechanism, which allows RunBot to autonomously learn by adapting its gait (described later).

The threshold Θ*_G_* of the ground contact sensor neurons is chosen to be 2.0 v following a test of the switch sensors, which showed that in a certain range the output voltage of the switch sensor is roughly proportional to the pressure on the foot bottom when touching the ground. The time constant of the motor neurons, τ (see Equation 4), is chosen as 10.0 ms, which is in the normal range of biological data. For the connection strengths *w_Z_* (see Equation 4) as denoted in Figure 6, we use: *w_NG_* ≥ Θ*_N_*, *w_NA_* − *w_NG_* ≥ Θ*_N_*, *w_NS_* − *w_NA_* − *w_NG_* ≥ Θ*_N_*, where *w_NG_* = weights of the synapses between the ground contact-sensor neurons and the motor neurons, *w_NA_* = weights of the synapses between the stretch receptors and the motor neurons, *w_NS_* = weights of the synapses between the angle sensor neurons and the motor neurons in the neuron modules of the joints, and Θ*_N_* = the threshold of the motor neurons (see Equation 5), which can be any positive value as long as the above conditions are satisfied. The function of these rules is to make sure that among all the neurons which contact the motor neurons, the angle sensor neurons have the first priority, while the stretch receptors have second priority, and the ground contact sensor neurons have lowest priority. So, we simply choose them as: Θ*_N_* = 5.0, *w_NG_* = 10.0, *w_NA_* = 15.0, *w_NS_* = 30.0 (compare Figure 6). A more detailed description of the neuronal controller and a discussion of stability issues of all parameters can be found in [33].

*Body control.* Body control of RunBot consists of two motor neurons (*N_E_* and *N_F_*) and one AS providing a reflex signal (see Figure 6). These neuron models are similar to those for leg control. The synaptic strengths of the connection structure are shown in Figure 6. This network is driven by the AS where its output *a_AS_* is modelled according to:

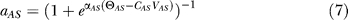
where *V_AS_* is the output voltage signal from the AS. Θ*_AS_* and *α_AS_* are the threshold and a positive constant which are set to 4.0 and 2.0, respectively. *C_AS_* is a positive amplification of the input signal set to 6.0.

The motor neurons (*N_E_*, *N_F_*), which directly modulate the motions of the UBC, have the same characteristic as the leg motor neurons (see Equations 4 and 5) but different parameters Θ*_N_*, *α_N_*, *D*, and *g*. We set Θ*_N_* of the extensor body–motor neuron to 0.75 and for the flexor to −0.75 and *α_N_* to 20.0, while *D* and *g* are both set to 1.0 (see Equation 6). Usually, for example when walking on a level floor, *N_F_* is activated to lean the body backward (see Figures 2A and 12) while *N_E_* is deactivated unless a strong signal from the AS drives its reflex (leaning the UBC forward); i.e., this signal excites *N_E_* while it inhibits *N_F_*. This situation happens only when RunBot falls backward; e.g., when RunBot tries to walk up a ramp.

### Adaptive neuronal controller with learning rule (postural reflex level).

To create adaptive behavior for walking on different terrains, an effective way is to let RunBot learn adapting its gait and controlling the posture of its UBC by itself. To this end, we apply a learning technique, which will finally allow RunBot to walk up a ramp and then continue again on a level floor. To sense a ramp when RunBot is making an approach, we use an IR sensor (see Figure 12), which requires some preprocessing before it can be used by our learning algorithm. Thus, in the following sections, we will describe the sensory preprocessing, followed by the details of the learning network together with the learning algorithm.

*Sensory preprocessing.* The raw infrared signals require preprocessing because they are too noisy due to RunBot's egomotion and because they arrive too early at the robot (hence before it reaches or leaves the ramp). To address these issues, we construct the neural preprocessing of the raw IR signal as a hysteresis element [66,67] using a single neural unit with a “supercritical” self-connection (*w_self_* > 4). It is modelled as a discrete-time nonspiking neuron, and its activation function is given by:

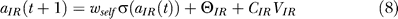
where *V_IR_* is the output voltage signal from the IR sensor, which is linearly mapped onto the interval [0, 1]. Θ*_IR_* is the threshold, and *C_IR_* represents a positive amplification factor of the input signal. The output of the neuron is given by the standard sigmoidal transfer function 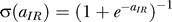 . To get an appropriate hysteresis, we set Θ*_IR_* = −3.2, *C_IR_* = 4.0, and *w_self_* = 4.8 (see Figure 13B). Note that the width of the hysteresis is proportional to the strength of the self-connection; i.e., the stronger the self-connection, the wider the hysteresis.

*Learning network and its effect—reflex avoidance learning.* In the following, we will describe our learning network, which enables RunBot to successfully perform the given task. To do so, its gait has to be changed as well as the posture of its UBC. The UBC is controlled by exciting or inhibiting *N_E_*, *N_F_* through sensory signals (described above).

We know from previous experiments [57] that a stable gait for upslope walking can be obtained by adjusting the following parameters. At the knee joints, the firing threshold 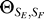 of neurons *S*_*E*_, *S_F_* has to be decreased; while at the hip joints, the firing threshold 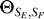 of neurons *S_E_*, *S_F_*, which also affects the stretch receptor neurons *A*, has to be increased, but the gain *g* of neurons *N_E_*, *N_F_*, has to be decreased. This leads to smaller steps, also observed in humans when climbing.

In our learning algorithm, the modification of all those parameters also common in human walking reflexes [68] will be controlled by two kinds of input signals: one is an *early* input (called predictive signal) and the other is a *later* input (called reflex signal). Here, we use the preprocessed IR signal as a predictive signal, while the AS signal serves as a reflex signal. Both sensory signals are provided to the learner neurons as shown in Figure 13.

At the beginning, the connections (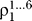 ) between the predictive signal and learner neurons converge with zero strengths. In this situation, parameters of the target neurons will be altered only by the reflex signal; i.e., the leaning reflex of the UBC together with the gait adaptation will be triggered by the AS signal as soon as RunBot falls. Hence, RunBot will begin walking up the ramp with a wrong set of gait parameters and an inappropriate posture of the UBC. Thus, it will eventually fall, leading to a signal at the AS, which will change RunBot's parameters—but too late (when it already lies on the ground). Due to learning the modifiable synapses, *ρ*_1_, which connects the predictive IR signal with the learner neurons, will grow. Consequently, after three to five falls during the learning phase, gait adaptation together with posture control of the UBC will finally be driven by the predictive IR-signal instead. Correspondingly, RunBot will adapt its gait together with leaning the UBC in time. The used learning algorithm has the property that learning will stop when the reflex signal is zero; i.e., when RunBot does not fall anymore [42]. On returning to flat terrain, the IR output will get small again and RunBot will change its locomotion back to normal for walking on a level floor. Note that the same circuitry and mechanisms can be used to learn different gaits for other given tasks, for example walking down a ramp.

Hence, the employed mechanism performs “reflex avoidance learning.” Synapses stop growing as soon as the new anticipatory reaction has been learnt and the reflex to the later signal is not triggered anymore. As mentioned above, the principle of reflex avoidance learning appears to be emulated by cerebellar function [29], albeit not by the same mechanisms as used here. The cerebellum rather seems to rely on an interplay between the mossy fiber to deep nucleus synapse and the parallel fiber to Purkinje cell synapse. The first seems to control the overall amplitude of a cerebellar response, the second the timing. The parallel fiber to Purkinje cell synapse does not seem to rely on STDP but rather it uses long-term depression to facilitate the reduction of Purkinje cell activity, leading to a release of the deep nucleus neurons to form inhibition and a rebound excitation. This possibly involves presynaptic mechanisms. This whole circuitry has been captured in a recent model by Hofstötter et al. [69]. Our learning rule operates at the single cell level using an STPD-like mechanism. This is necessary to achieve the required efficiency for real-time learning. Hence the same principle (reflex avoidance) is used here but with a different implementation, very much focusing on algorithmic efficiency.

*Learning algorithm.* In general, each learner neuron *L_n_* requires two input signals *u*_0_ and *u*_1_ with synaptic weights *ρ*_0,1_. Here, we use the AS and the preprocessed IR signals as *u*_0_ and *u*_1_, respectively.

Furthermore, we initially set 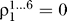 and 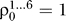 . Only *ρ*_1_ is allowed to change through plasticity. The output activity *v* of *L_n_* is given by:

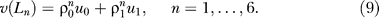



Note, since *v* is defined by weights and input strengths, we will—after learning—receive differently strong outputs for differently strong input signals IR (signal *u*_1_). Hence, after having learned a steep slope, less steep slopes will drive the output less, leading to smaller parameter changes and incomplete leaning of the body, which is the appropriate behavior, in this case preventing a fall (not shown).

We use a differential Hebbian learning rule (ISO-learning, [42]) for the weight change of 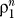 given by:

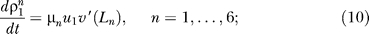
where *v*′(*L_n_*) is the temporal derivate and *μ_n_* the learning rate. It is independently set for each learner neuron, which will define the desired equilibrium point (*μ_1_* = 10, *μ_2_* = 7.0, *μ_3_* = 10.5, *μ_4_* = 0.14, *μ_5_* = 3.0, *μ_6_* = 10.0). One could consider *μ* as the susceptibility for a synaptic change, which in a biological agent will be defined by its evolutionary development, which determines the agent's ability to learn a certain task. How and if these values could also be influenced (possibly by mechanisms of meta-plasticity), changing learning susceptibility, goes beyond the scope of this article.

Our learning rule is based on differential Hebbian learning [70], described in detail in [42]. Hence, this form of plasticity depends on the timing of correlated signals and thereby compares with STDP [41,71]. In neurons with multiple inputs, such a mechanism can be used to alter the synaptic strengths according to the order of the arriving inputs. Note that neuronal time scales for STDP do not match the much longer time scales required here. There are mechanisms discussed in the literature to address this problem [72]. In the context of the current study, we are, however, not concerned with this, and we are using Equations 9 and 10 directly. As a consequence of this rule, the modifiable synapses *ρ*_1_ will get strengthened if the predictive signal *u*_1_ is followed by the reflex input *u*_0_, where the reflex drives the neuron into firing. This rule will lead to weight stabilization as soon as *u*_0_ = 0 [42]; hence, when the reflex has successfully been avoided. As a result, we obtain behavioral and synaptic stability at the same time without any additional weight-control mechanisms.

The output of each learner neuron *v*(*L_n_*) is directly fed to its target neuron in the network. The connection structure together with its synaptic polarity *ζ* is shown in Figure 13. To control the UBC, we directly use the average firing rate of the learner neuron *v*(*L*_1_) to drive the body motor neurons *N_E_* and *N_F_*. Once the learner neuron *L*_1_ gets active, it will inhibit *N_F_*, while *N_E_* will be activated. As a result, the UBC will lean forward. As described above, changing the gait of RunBot is achieved by controlling the values of the output gain of the leg motor neurons *g* and the firing threshold Θ of sensor neurons using the firing rate of learner neurons. To change a threshold, one can simply redefine the input signal *I* of the sensor neurons (*A_L_*, *A_R_*, *S_E_*, *S_F_*) presented in Equations 2 and 3 as:

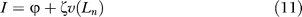
where *I* is the input summation of the real time angular position *φ* and the average firing rate of a learner neuron *v*(*L_n_*), and *ζ* is the connection polarity learner and target neuron (see Figure 13).

To change the output gain of the hip motor neurons, we need to divide or multiply. Hence, the learner neuron *L*_4_ performs divisive (shunting) inhibition [73], which in a real neuron is commonly generated by the influence of *GABA_A_* on chloride channels ([74,75], but see [76]). Thus, the gain of *N_E_* and *N_F_* is affected by divisive inhibition, defined by:

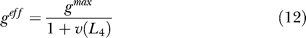
where *g^max^* is the maximum motor gain which is set to 2.2 for an optimal walking speed. Note that *g^max^* is proportional to the walking speed and it can be set to up to 3.0, beyond which the motors are damaged.

## Supporting Information

Video S1RunBot Can Perform Self-Stabilization When Changing Speed on the FlyIn this situation, we immediately switch from a slower walking speed of 39 cm/s (≈1.7 leg-length/s) to a faster one of 73 cm/s (≈3.17 leg-length/s). This has been achieved by abruptly and strongly changing two parameters: 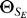 and *g_H_*. Self-stabilization reflects the cooperation between the mechanical properties and the neuronal control. Furthermore, it shows that RunBot's neuronal controller is robust to quite drastic and immediate parameter variations. Note that the real time data of the joint angles recorded during walking and changing speed on the fly is presented in Figure 5. (http://www.nld.ds.mpg.de/~poramate/RUNBOT/ManoonpongMovieS1.mpeg)
(4.2 MPG).

Video S2 RunBot Learns to Walk up an 8° Ramp Where *g^max^* Is Set to 2.2 It can achieve this after three falls. Consequently, it can autonomously adapt its gait to walk on different terrains, i.e., walking from a level floor to a ramp and then again to a level floor. (http://www.nld.ds.mpg.de/~poramate/RUNBOT/ManoonpongMovieS2.mpeg)
(5.1 MPG).

## Abbreviations

AEA: anterior extreme angle
AS: accelerometer sensor, CPG, central pattern generator
IR: infrared
STDP: spike timing–dependent plasticity
UBC: upper body component
