# Correction: Adaptive, Fast Walking in a Biped Robot under Neuronal Control and Learning

**DOI:** 10.1371/journal.pcbi.0030191

**Published:** 2007-09-28

**Authors:** Poramate Manoonpong, Tao Geng, Tomas Kulvicius, Bernd Porr, Florentin Wörgötter

doi:10.1371/journal.pcbi.0030134

In Figure 1F, the number 0.24 should be 2.4 instead. The incorrect Froude number given for human walking (0.24) corresponds to 1.5m/s, which is closer to the preferred speed of human walking. The correct number now given (2.4) corresponds to a speed of about 4.6m/s.

The incorrect Figure 1 is found at: doi:10.1371/journal.pcbi.0030134.g001.

